# Inhibition of Advanced Glycation End-Product Formation by High Antioxidant-Leveled Spices Commonly Used in European Cuisine

**DOI:** 10.3390/antiox8040100

**Published:** 2019-04-15

**Authors:** Małgorzata Starowicz, Henryk Zieliński

**Affiliations:** Department of Chemistry and Biodynamics of Food, Institute of Animal Reproduction and Food Research of Polish Academy of Sciences, Tuwima 10, 10-748 Olsztyn, Poland; h.zielinski@pan.olsztyn.pl

**Keywords:** spices, herbs, antiglycation activity, AGEs, antioxidants, total phenolic content, natural inhibitors

## Abstract

Spices and herbs, as good sources of polyphenols, could be strong inhibitors of advanced glycation end-product (AGE) formation. The aim of this research was to measure the ability of various spices to inhibit AGEs and to study the correlation of AGE inhibition with total phenolic (TP) content and antioxidant capacity. Fourteen spices commonly used in European cuisine were extracted with a 50% ethanol solution, and their water and total phenolic contents and antioxidant capacities were examined. Antioxidant capacity was evaluated using three methods: (1) Measurement of the radical scavenging ability of 2,2’-azino-bis-(3-ethylbenzothiazoline-6-sulphonic acid) (ABTS) and (2) 2,2-diphenyl-1-picrylhydrazyl (DPPH●); and (3) photochemiluminescence (PCL) assay. Antiglycation properties were studied in vivo using two model systems: Bovine serum albumin-glucose (BSA-glucose) and bovine serum albumin-methylglyoxal (BSA-MGO). The most potent glycation inhibitors, according to the BSA-MGO assay, were star anise (88%), cinnamon (85%), allspice (81%), and cloves (79%), whereas in the BSA-glucose measurement, oregano was noted to be a very effective inhibitor of the glycation process. The ability to inhibit glycation was highly correlated with TP values in the BSA-MGO and BSA-glucose assay (*r* = 0.84 and 0.76, respectively). Our research showed the high antiglycation ability of cinnamon, cloves, and allspice, and we suggest, for the first time, that anise could also be considered a good glycation inhibitor.

## 1. Introduction

Glycation is a reaction that leads to the formation of irreversible structures called advanced glycation end-products (AGEs). A high tissue concentration of AGEs can initiate actions that lead to various disorders and their associated complications, such as Alzheimer’s disease, atherosclerosis, diabetes, kidney disease, and chronic heart failure [1,2]. It has been shown that extracts from some plants, which are a rich source of phenolic compounds, can inhibit AGE formation through their strong antioxidant properties [3]. For example, quercetin isolated from *Rumex japonicus* was reported to be a good inhibitor of AGEs [4]. Further studies have shown that caffeic and chlorogenic acid extracted from *Ilex paraguariensis* limit the glycation process through the blockage of glycation precursors, such as methylglyoxal (MGO) [5]. Among the dicarbonyl intermediates, MGO is known to be a very reactive precursor of AGEs [6]. Therefore, it has received considerable attention as a mediator of AGE formation, and is known to react with lysine, arginine, and cysteine residues to form glycosylamine protein crosslinks [6]. Our study focused on D-glucose because it is the main glycating sugar and is present at the highest concentration in comparison to other sugars. Accordingly, to measure the antiglycation ability, the bovine serum albumin–methylglyoxal (BSA-MGO) system was used as the marker of the middle stage of formation of oxidative cleavage products, and the BSA-glucose model was used to describe the final stage of AGE formation [7]. For example, a very high antiglycation ability has been observed for spices that are characteristic of Mexican cuisine, among which *Piper auritum* and *Origanum majorana* are the most active antiglycative spices [8]. A previous study revealed that even *Piper auritum,* with a 50% inhibitory concentration (IC_50_) value of 20.07 µg mL^−1^, is more effective to inhibit glycation than the positive control aminoguanidine (28.2 IC_50_) [8]. The authors of this publication did not observe strong evidence that high AGE inhibition potency is related to high antioxidant activity in every spice tested. However, in other studies, a strong correlation was demonstrated between the inhibitory effect on AGE formation and both the flavonoid content and the antioxidant capacity in extracts of *Scutellaria alpina* L. and *S. altissima* (AGEs vs. flavonoid content and AGEs vs. Ferric Reducing Antioxidant Power (FRAP); correlation coefficients of 0.99 each) [9]. Furthermore, a strong correlation was observed between the total phenolic content (TPC) and AGE inhibition in the BSA-glucose model (correlation coefficient of 0.9), and between 2,2-diphenyl-1-picrylhydrazyl (DPPH) and AGE inhibition (correlation coefficient of 0.6) when tested in 10 typical spices and herbs (garlic, thyme, ginger, parsley, mint, coriander, curry plant, turmeric, onion, and Welsh onion) [10]. Thus, research on the relationships between AGE inhibition and other factors is not conclusive.

For these reasons, the aim of this study was to evaluate the AGE inhibition of selected spices and herbs that are characteristic of European cuisine. The correlations of the AGE inhibition with both the total phenolic (TP) content and the antioxidant capacity were investigated using 2,2’-azino-bis-(3-ethylbenzothiazoline-6-sulphonic acid) (ABTS●^+^), DPPH●, and superoxide anion radicals (O_2_●^−)^ by means of a photochemiluminescence (PCL) method. The analyses were performed on selected spices, namely, anise, cumin, coriander, fennel, parsley, cardamom, ginger, cloves, allspice, black and white pepper, thyme, oregano, star anise, nutmeg, and cinnamon. Evaluation of the antiglycation properties of the selected spices was performed via in vitro studies using two model systems: BSA-MGO and BSA-glucose. In both experiments, the results were compared to aminoguanidine, the reference substance (positive control).

## 2. Materials and Methods

### 2.1. Chemicals

Aminoguanidine (AG), 2,2’-azino-bis(3-ethylbenzothiazoline-6-sulphonic acid) (ABTS), 2,2-diphenyl-1-picrylhydrazyl (DPPH), 6-hydroxy-2,5,7,8-tetramethylchroman-2-carboxylic acid (Trolox), D-glucose, sodium azide, bovine serum albumin (BSA), and methylglyoxal (MGO) were purchased from Sigma (Sigma Chemical Co., St. Louis, MO, USA). Folin–Ciocalteu reagent, sodium carbonate, and ethanol (HPLC purity) were provided by POCh (Gliwice, Poland). Water was purified with the Mili-Q-system (Millipore, Bedford, Massachusetts, USA). PCL assay kit was purchased from Analytical Jena (Jena, Germany). To prepare the phosphate buffer (0.1 M, pH 7.4), monosodium phosphate and dipotassium phosphate were mixed at the appropriate ratio.

### 2.2. Plant Material Extraction Procedure

The fourteen herbs and spices used were purchased from local supermarkets in Poland and Slovakia. The herbs and spices included anise (*Pimpinella anisum* L.), allspice (*Pimenta dioica* L.), star anise (*Illicium anisatum* L.), black and white pepper (*Piper nigrum* L.), cardamom (*Elettaria cardamomum* L.), cinnamon (*Cinnamomum verum* J.), cloves (*Syzygium aromaticum* L.), ginger (*Zingiber officinale* Rosc.), nutmeg (*Myristica fragrans* H.), oregano (*Origanum vulgare* L.), parsley (*Petroselinum crispum* Mill.), star anise (*Illicium verum* L.), and thyme (*Thymus vulgaris* L.), all of which are commonly used in European cuisine.

Dried and homogenized spices (0.1 g for each spice) were placed into 5 mL vials, and 1 mL of aqueous ethanol solvent (1:1, *v*/*v*) was added to each. Then, the mixtures were vortexed for 1 min and extracted in an ultrasonic bath for 1 min. In the next step, the mixtures were centrifuged for 10 min at 5000× *g* at 4 °C. After that, the resultant supernatants were transferred to other vials and the spice residues were re-suspended in fresh extraction solvent. These steps were repeated 5 times until 5 mL of each spice supernatant had been extracted. Three independent extractions were completed for each spice. The ethanolic extracts were kept at −20 °C prior to further analysis for total phenolic (TP) content and antioxidant capacity.

### 2.3. Determination of Total Phenolic (TP) Content

The total phenol contents of the spice extracts were analyzed according to the method proposed by Magalhães’ group [11]. The absorbance at 750 nm was measured by the Infinite M1000 PRO microplate reader (Tecan Group Ltd., Switzerland), and measurements were performed in triplicate and are presented as milligrams of gallic acid equivalent per gram of dry matter (mg Gallic acid equivalent (GAE) g^−1^ DM). 

### 2.4. Determination of Antioxidant Activity

The antioxidant capacity was established in terms of DPPH and the superoxide anion radical scavenging ability (photosensitized chemiluminescence as PCL assay) of ethanolic extracts (1:1, *v*/*v*) of spices and herbs. The DPPH measurement method was recently described in detail by Magalhães [11] and the PCL method was performed in accordance with Popov and Lewin [12]. The antioxidant capacity was calculated as µmol Trolox equivalents per gram of dry matter [µmol Trolox g^−1^ DM].

### 2.5. Advanced Glycation End Products (AGEs) Assay

The AGEs assay uses fluorescence spectroscopy to monitor the inhibitory effect exerted against glycation in the presence or absence of a substance or an extract using a reaction model system. The anti-glycation activity of herb and spice extracts was determined by two model systems: BSA-MGO and BSA-glucose. The BSA-glucose and BSA-MGO models were adapted from Lunceford and Gugliucci [13] and Szawara-Nowak et al. [14], with slight modifications to compare the antiglycation abilities of different spice and herb extracts. First, approximately 300 mg of dried spices or herbs was weighed into plastic vials, and 1 mL of 50% EtOH solution was added. The mixture was shaken for 2 h at 25 °C with a Thermomixer C (Eppendorf, Hamburg, Germany) and centrifuged for 10 min at 16,000× *g* (Centrifuge Eppendorf 5415R, Hamburg, Germany). Then, the supernatant was collected, and the same extraction step was repeated with 1 h of shaking. The supernatants were pooled and evaporated until dry. The dried residue was made up in 1 mL of phosphate buffer (pH 7.4).

#### 2.5.1. BSA-Glucose Model System Preparation

BSA (10 mg mL^−1^) and glucose (90 mg mL^−1^) were dissolved separately in phosphate buffer (pH 7.4). Then, 1 mL of the previously prepared spice or herb extracts was mixed with 1 mL of BSA and 1 mL of glucose solution in a 5 mL polypropylene test tube. A blank was prepared with 1 mL of phosphate buffer, 1 mL of BSA solution, and 1 mL of glucose solution. A positive control was prepared using 1 mL of AG solution (1 mol L^−1^) mixed with 1 mL of BSA and 1 mL of glucose. The tested solution also contained 0.01% of NaN_3_ to prevent microbe development. The tubes were caped and incubated for 72 h at 37 °C, in the dark in a temperature-controlled incubator.

#### 2.5.2. BSA-MGO Model System Preparation

BSA (2 mg mL^−1^) and MGO (400 mg mL^−1^) were dissolved separately in phosphate buffer (pH 7.4). The rest of the procedure was the same as that for the BSA-glucose model. The incubation time was extended to 7 days at 37 °C in darkness.

#### 2.5.3. Measurement of AGE Fluorescence

The fluorescence of AGEs was measured after an appropriate incubation procedure was conducted at excitation and emission wavelengths of 360 and 420 nm for BSA-glucose and 340 nm and 420 nm for BSA-MGO using an Infinite M1000 PRO microplate reader (Tecan, Switzerland). Triplicate samples were run for each set, and the percent inhibition of AGE formation by spice and herbs extracts was calculated, using AG as a positive control. The following equation was used (FI: fluorescence intensity):

Inhibition [%] = {1 − [(FI of extract)/(FI of blank)]} × 100(1)

#### 2.5.4. Statistical Analysis

The results are given as the mean ± SD of three independent measurements. Statistical one-way analysis of variance (ANOVA) using the Fisher’s Least Significant Difference (LSD) test (*p* ≤ 0.05) was performed for all obtained results. Pearson correlation coefficients and Principal Component Analysis (PCA) were determined using statistical software for Excel (XLSTAT, ver. 19.01, Addinsoft, CA, USA).

## 3. Results and Discussion

### 3.1. TP Content and Antioxidant Capacity Determination of Selected Herbs and Spices

The TP content and antioxidant capacity data are shown in Table 1. The same solvent (ethanol: water, 1:1 *v*/*v*), which was proven to be the best solvent in our previous research [15], was used for the extraction of phenolic compounds from all spices to evaluate their antioxidant and anti-glycation activities. This was required to maintain the proper link between the TPC contents and functional markers to allow a correlation analysis to be performed. The choice of solvent and extraction method was crucial to allow the profiles of all compounds with phenolic functions to be assessed. It is well-known that spices are very rich in chemical constituents with an antioxidant capacity, but this does not mean that all of these compounds can be fully extracted by aqueous ethanol. About 1% of oregano, thyme, cloves, and anise is terpenoid compounds with phenolic functions, and other extraction methods are required to provide their full chemical profiles. Such methods include obtaining the spices’ essential oils and oleoresins by hydrodistillation or high-pressure CO_2_ extraction [16]. On the other hand, simple water extraction of the TPC was described by Lu et al. [16] as being a sufficient to show the high positive correlation between TPC and the antioxidant capacity of spices commonly consumed in China.

Information on the antioxidant activity of chemical constituents in spices is scarce. For example, anise contains trans-anethole, estragole, and anise ketone, as well as other components in minor concentrations, such as β-caryophyllene, anisaldehyde, anisic acid, linalool, limonene, α-pinene, acetaldehyde, p-cresol, creosol, hydroquinine, β-farnasene, γ-himachalene, and ar-curcumene. Cloves are rich in eugenol, eugenol acetate, β-caryophyllene, α-cububene, α-copaene, isoeugenol, nerolidol, and farnesol [17]. Therefore, it is necessary to consider the activity of the phenolics from spices as cumulative and synergistic, as described by Markova, who tested the use of spices as an acrylamide mitigation strategy in spices-enriched cookies [18].

The TP content ranged from 2.5 to 190.7 mg of GAE g^−1^ DM. The highest TP content was observed in extracts of anise, allspice, and cinnamon, followed by cloves. TP values were lower in extracts of black pepper, cumin, oregano, and thyme, and the spices with the lowest TP contents were parsley, nutmeg, ginger, star anise, and white pepper. These findings are in accordance with results of Assefa, Keum, and Saini [19], who found the highest TP contents in cloves, cinnamon, and allspice and low and medium TPC in white pepper, ginger, parsley, star anise, and nutmeg. A significantly higher TPC value was recorded for black pepper in comparison to white pepper (17 times higher). The difference might be due to the cultivation steps. Berries of white pepper are collected at an unripe stage, whereas black pepper berries are collected when fully ripened [20]. Additionally, a comparative study of black and white pepper revealed that the concentrations of hydrolyzed and non-hydrolyzed polyphenolic compounds are both higher in black pepper [20]. It is hard to determine the spices and herbs with the highest TPC, as this might be linked to differences in genotoxic and environmental influences. This problem was pointed out previously by Wojdyło, Oszmiański, and Czemerys [21], regarding the antioxidant characterization of spices from the Lamiaceace family. They measured high polyphenolic contents in spices from the Lamiaceace family, for example, oregano and sage, but they did not measure the very high contents found in other studies. In our investigation, oregano and thyme were not classified as part of the group with the highest TPC values. The TPC describes the overall content of polyphenolic compounds. Selected molecules that might contribute to TPC are apigenin in parsley, quercetin in cloves, black and white pepper, caffeic acid in oregano, caffeic and neochlorogenic acids in thyme, ferulic acid in nutmeg, and sinapic and cinnamic acids in cinnamon [21,22,23]. These biologically active compounds might have great influences on antioxidant activity.

According to differing characteristics of biologically active compounds, more than one method is required to verify the antioxidant activity. Therefore, the antioxidant activity of ethanol extracts of selected spices and herbs was verified by three methods: ABTS and DPPH assays, and antioxidative capacity of lipid-soluble (ACL) compounds measured by PCL (Table 1). The scavenging ability of the ABTS cation radical ranged from 39.4 (cumin and ginger) to 2071.1 (cloves) µmol TE g^−1^ DM. Moreover, the highest ABTS values were also measured in cinnamon and allspice. These findings are in accordance with Assefa, Keum, and Saini [19], who also described clove, cinnamon and allspice as being the most active scavengers against the ABTS cation radical. In our study, star anise and nutmeg were identified as being highly antioxidant plant materials. The measurement of antioxidant activity by DPPH showed the same relationships as the ABTS assay. However, oregano and cumin (93.4% and 93.2% inhibition, respectively) exhibited the highest antioxidant activity, followed by cinnamon (90.7%), cloves (88.3%), and thyme (88.1%). Embuscado also reported a high DPPH inhibition ability for oregano, thyme, and clove, despite the fact that extraction was done using hot water [24]. With the PCL ACL method, cloves, cinnamon, and allspice were shown to be the most active scavengers of O_2_●^−^, followed by oregano, thyme, ginger, cumin, nutmeg, and black pepper. The group of spices with the lowest scavenging ability included star anise, white pepper, anise, parsley, and cardamom. This could mean that higher concentrations of lipid-soluble molecules can be found in some spices, and these have a strong contribution to the overall antioxidant activity. For example, the PCL ACL value for cinnamon (512.0 µmol TE g^−1^ DM) was significantly higher than that obtained by the water-soluble PCL method (177.4 µmol TE g^−1^ DM) in our previous study [15]. However, the PCL measurements of antioxidative capacity of water-soluble (ACW) compounds for cloves (926.8 µmol TE g^−1^ DM) and allspice (280.2 µmol TE g^−1^ DM) are higher than the ACL values of 896.5 and 143.7 µmol TE g^−1^ DM, respectively [15]. In all methods, black pepper was observed to be a more effective radical scavenger than white pepper. Higher antioxidant values for black pepper were also noted in data collected by Shahidi and Ambigaipalan [25]. They explained that black peppercorn is a richer source of biologically active compounds, such as piperine and its isomers, in comparison with white pepper; therefore, its antioxidant activity is higher.

### 3.2. Results of AGE Inhibitory Ability Among Selected Herbs and Spices

**Figure 1:**
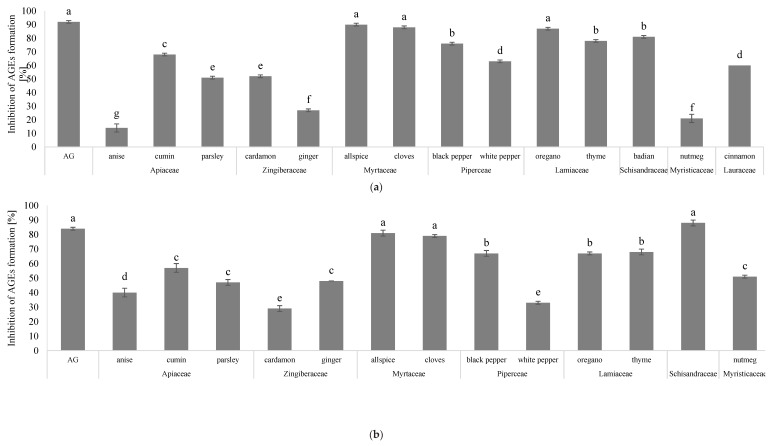
The ability of spices and herbs’ extracts to inhibit AGEs formation measured in (**a**) BSA-glucose and (**b**) BSA-MGO model systems. AG: aminoguanidine; AGEs: Advanced Glycation End- products.

In our investigation, we found positive influences of spice and herb extracts on the inhibition of AGEs, as monitored by the BSA-glucose system (Figure 1a). The positive control, aminoguanidine (AG), highly inhibited formation of fluorescent AGEs (92%). Extracts of allspice (90%), cloves (88%), oregano (87%), and star anise (81%) were shown to be the most effective glycation inhibitors, whereas ginger, nutmeg, and anise possess the lowest ability to inhibit AGE formation. The rank of AGE inhibition ability was as follows: allspice > cloves > oregano > star anise > thyme > black pepper > cumin > white pepper > cinnamon > cardamom > parsley > ginger > nutmeg > anise. The high antiglycation ability of cloves and allspice was proven by Dearlove et al. [26]. We also noted that the inhibitory level for white pepper was over two times lower than that of black pepper (33% and 67%, respectively). It can be assumed that this might be linked to the preparation procedure of white pepper. White pepper is the soaked and dried version of black pepper. It is suggested that during this procedure, the active components might be degraded or lost. In this study, we also found that the extracts of spices and herbs inhibited the formation of AGEs in a BSA-MGO system. This positive effect might be related to blockage of the conversion of dicarbonyl intermediates to AGEs. The AGE inhibitory properties shown by the BSA-MGO system are presented in Figure 1b. The most effective AGE formation inhibition was shown by extracts of star anise (88%) and cinnamon (85%), followed by allspice (81%) and cloves (79%). In the BSA-MGO system, AG inhibited the formation of fluorescent AGEs by 84%. Star anise and cinnamon showed slightly higher inhibition potential than AG; however, the difference was not significant. The high antiglycation potential of cinnamon is likely to be related to the presence of procyanidin B2 or cinnamon acid and their derivatives, which have been described as very good inhibitors of AGE formation [3,27]. Previous results for cinnamon bark showed higher inhibition than was seen in our study, which might be related to differences in extraction procedures. Moreover, our study is in agreement with research by Kazeem, who also observed that the antiglycation ability of pepper is significantly higher than that of nutmeg and ginger [28].

The mechanism by which polyphenols inhibit AGE is a crucial part of studies about AGE-related disease prevention; however, it still remains unclear [29]. In a study by Sadowska-Bartosz et al. [30], the authors pointed out that the mechanism of glycosylation inhibition by different polyphenols is related to the presence of various sugars. Flavonoids have the highest ability to prevent glycosylation. It has been said that flavonoids bind to proteins, and this might be a protective action against AGE formation. The same inhibitory mechanism was described for the polyphenol family by Bhattacherjee and Datta [31]. In their study, they found syringic acid to a better AGE inhibitor than chlorogenic acid because it is a smaller molecule and therefore has higher accessibility to bind to amino groups of lysine [32]. Therefore, syringic acid is also a competitive inhibitor of glucose. Another study described high aggregation inhibition and AGE formation with epigallocatechin-3-gallate [32]. This is mainly due to the building of stable colloid between epigallocatechin-3-gallate and albumin, which may protect against any other intermolecular interactions [32]. Positive antiglycation ability was also found in the Korean herb *Houttuynia cordata* [33]. The polyphenols quercetin and rutin were shown to be mainly responsible for the high inhibitory properties of this herb. Therefore, it was said that these polyphenols do not bind to proteins, but rather, react through the formation of adducts with MGO [34]. The AGE inhibitory activity of *Houttuynia cordata* was shown to be 2–3 times higher than that of aminoguanidine; however, Yoon and Shim [33] incubated samples for three weeks, so the time of incubation might have led to the better inhibitory activity in this case.

### 3.3. Correlation Study and Principal Component Analysis (PCA)

According to Pearson’s correlation test (presented in Table 2), a high positive correlation was obtained between the BSA-glucose and BSA-MGO model systems (*p* < 0.01, *r* = 0.924), and between BSA-MGO and TPC (*p* < 0.01, *r* = 0.861). Medium positive correlations were obtained between BSA-MGO and PCL (*p* < 0.01, *r* = 0.716), BSA-glucose and TPC (*p* < 0.01, *r* = 0.714), and BSA-glucose and PCL (*p* < 0.01, *r* = 0.746). Weaker correlation coefficients were calculated for BSA-MGO and ABTS (*p* < 0.01, *r* = 0.621), BSA-glucose and ABTS (*p* < 0.01, *r* = 0.571), BSA-MGO and DPPH (*p* < 0.01, *r* = 0.526), and BSA-glucose and ABTS (*p* < 0.01, *r* = 0.498). A stronger correlation was demonstrated between antiglycation and antioxidant activities of our herbs and spices than in wild berries [35]. Our results are with agreement with Deetee et al. [36], who also demonstrated high correlation coefficient values in tea extracts between BSA-glucose, TPC, and antioxidant activity, as measured by ABTS and FRAP.

**Figure 2:**
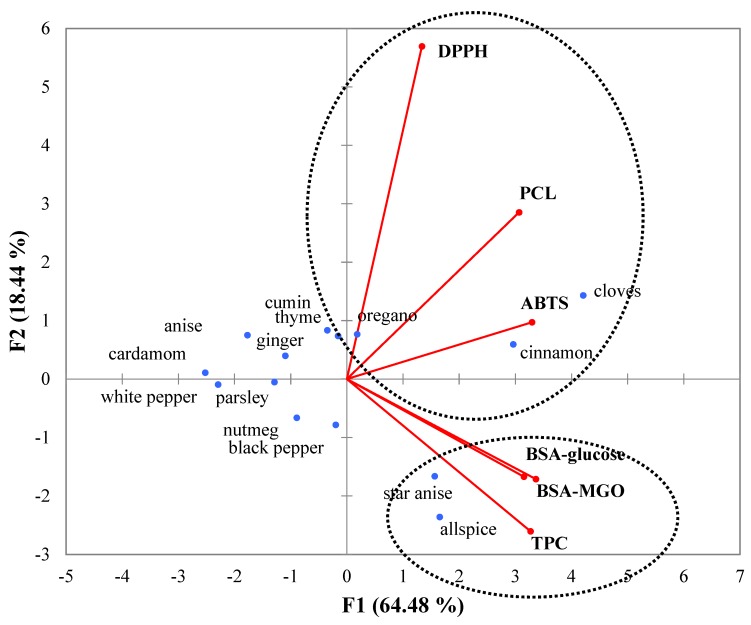
The score plots of analyzed spices.

The PCA yielded two Principal Components (PCs) with Eigen values >1 (Figure 2), accounting for 82.92% of the total variation in the dataset. The first component (F1) was mainly attributed to BSA-glucose, then BSA-MGO, and finally TPC, whereas the second component (F2) was described as being due to antioxidant activity, as determined by DPPH, PCL ACL, and ABTS assays. TPC correlated strongly with BSA-glucose and BSA-MGO. This might suggest that phenolic compounds are mostly responsible for the antiglycation properties of spice extracts, whereas other compounds that also have antioxidant potential are partly responsible for the glycation inhibitory activity. The best agreement between antiglycation and TPC was reported for allspice and star anise. Good agreement was observed for cloves, cinnamon, and oregano.

The positive correlation between AGE assays, total phenols, and antioxidant capacity shows that the high inhibitory activity of spices is due to their rich spectra of molecules with high antioxidant potential. Therefore, these selected spices can be recommended for usage in future applications in other foodstuffs, not only as a flavor ingredient but also as a nutrient.

## 4. Conclusions

The results of this study support the view that spices are promising AGE inhibitors. Among the spices explored in the present investigation, the top five spices with the greatest antiglycation potential, as measured by the BSA-glucose model, were allspice, cloves, oregano, star anise, and thyme, and the top five spices measured by the BSA-MGO model were star anise, cinnamon, allspice, cloves, and thyme. Therefore, allspice, cloves, cinnamon, star anise, oregano, and thyme can be recommended for use in functional bakery products or other foodstuffs, not only as a flavor ingredient but also as a nutrient. Few studies have described the application of polyphenols to reduce AGEs in food products [36,37]. Furthermore, these spices and their phenolic compounds can be used as ingredients to prevent glycation molecule formulation in food products and to minimize the consumption of food AGEs and their accumulation in the human body, which might help to prevent glycation-related diseases [38]. This information is important as it adds to the knowledge about AGE formation in food products and it can be used for functional product formulation and the promotion of zero waste production using products such as coffee silverskin, fruit and vegetable seeds, and white grape skin as a by-product of winemaking [39,40,41].

## Figures and Tables

**Table 1:** Total phenolic (TP) content and antioxidant capacity measured against ABTS●^+^, DPPH● and O_2_●^−^ radicals (PCL assay) in spices and herbs divided by their origin.

No.	Spices	Botanical Name	Total Phenolics (mg of GAE g^−1^ DM)	Antioxidant Capacity		
				ABTS (μmol TE g^−1^ DM)	DPPH (% of Inhibition)	PCL ACL (μmol TE g^−1^ DM)
1	Anise	*Pimpinella anisum* L.	8.2 ± 0.4 ^e^	61.6 ± 0.2 ^i^	65.9 ± 5.9 ^c^	21.8 ± 0.2 ^j^
2	Cumin	*Cuminum cyminum* L.	28.1 ± 2.3 ^d^	39.4 ± 2.2 ^k^	93.4 ± 3.2 ^a^	46.0 ± 2.6 ^f^
3	Parsley	*Petroselinum crispum* Mill.	13.5 ± 0.5 ^d^	40.3 ± 1.8 ^k^	48.3 ± 0.7 ^d^	18.3 ± 0.8 ^k^
4	Cardamom	*Elettaria cardamomum* L.	3.3 ± 0.1 ^f^	46.1 ± 2.1 ^j^	31.7 ± 0.1 ^e^	13.7 ± 0.4 ^l^
5	Ginger	*Zingiber officinale* Rosc.	11.3 ± 0.1 ^d^	39.4 ± 0.8 ^k^	60.7 ± 2.8 ^c^	92.5 ± 7.6 ^e^
6	Allspice	*Pimenta dioica* L.	183.9 ± 1.3 ^a^	718.8 ± 10.8 ^e^	6.6 ± 1.6 ^g^	143.7 ± 8.8 ^c^
7	Cloves	*Syzygium aromaticum* L.	156.7 ± 3.5 ^b^	2071.1 ± 75.5 ^a^	88.3 ± 2.4 ^b^	896.5 ± 4.3 ^a^
8	Black pepper	*Piper nigrum L.*	43.1 ± 0.1 ^c^	43.1 ± 0.1 ^k^	43.0 ± 2.3 ^d^	33.5 ± 0.4 ^g^
9	White pepper	*Piper nigrum L.*	2.5 ± 0.1 ^g^	83.0 ± 2.3 ^h^	27.2 ± 3.3 ^f^	23.6 ± 0.1 ^i^
10	Oregano	*Origanum vulgare* L.	26.6 ± 6.4 ^d^	106.8 ± 0.9 ^f^	93.2 ± 0.9 ^a^	116.0 ± 5.5 ^d^
11	Thyme	*Thymus vulgaris* L.	24.8 ± 2.7 ^d^	94.1 ± 5.1 ^g^	88.1 ± 2.2 ^b^	94.3 ± 13.0 ^e^
12	Star anise	*Illicum verum* L.	190.7 ± 17.5 ^a^	500.4 ± 14.7 ^d^	43.2 ± 3.7 ^d^	24.8 ± 0.8 ^h^
13	Nutmeg	*Myristica fragrans* H.	11.8 ± 0.6 ^e^	289.8 ± 14.1 ^e^	27.1 ± 4.0 ^f^	45.1 ± 6.1 ^f^
14	Cinnamon	*Cinnamomum verum* J.	180.6 ± 14.2 ^a^	1119.9 ± 199.2 ^b^	90.7 ± 0.1 ^b^	512.0 ± 19.3 ^b^

Values are means ± standard deviation (*n* = 3). Values in each column with different small superscript letters are significantly different (*p* < 0.05). GAE: Gallic Acid Equivalent; DM: dry matter; ABTS: 2,2’-azino-bis(3-ethylbenzothiazoline-6-sulphonic acid); DPPH: 2,2-diphenyl-1-picrylhydrazyl; PCL ACL: antioxidative capacity of lipid-soluble (ACL) compounds measured by photochemiluminescence assay TE: Trolox Equivalent.

**Table 2:** Pearson’s correlation coefficient between BSA-MGO, BSA-glucose, and TPC, and the antioxidant activity (ABTS, DPPH and PCL) of spices.

	BSA-MGO	BSA-glucose	TPC	ABTS	DPPH	PCL ACL
BSA-MGO	1.000					
BSA-glucose	0.924	1.000				
TPC	0.861	0.714	1.000			
ABTS	0.621	0.571	0.752	1.000		
DPPH	0.526	0.498	0.314	0.417	1.000	
PCL ACL	0.716	0.746	0.762	0.642	0.471	1.000

BSA-MGO: Bovine Serum Albumin- Methylglyoxal; BSA- glucose: Bovine Serum Albumin- glucose; TPC: Total Phenolic Compounds.
